# A simple method for construction of *pir^+ ^*Enterobacterial hosts for maintenance of R6K replicon plasmids

**DOI:** 10.1186/1756-0500-5-157

**Published:** 2012-03-20

**Authors:** Brian H Kvitko, Steven Bruckbauer, John Prucha, Ian McMillan, Erin J Breland, Stephanie Lehman, Katie Mladinich, Kyoung-Hee Choi, RoxAnn Karkhoff-Schweizer, Herbert P Schweizer

**Affiliations:** 1 Department of Microbiology, Immunology and Pathology, Colorado State University, IDRC at Foothills Campus, 0922 Campus Delivery, Fort Collins, CO 80523, USA; 2 Department of Oral Microbiology, College of Dentistry, Wonkwang University, Iksan 570-749, South Korea

## Abstract

**Background:**

The R6K replicon is one of the best studied bacterial plasmid replicons. Replication of the R6K plasmid and derivatives harboring its γ origin of replication (*ori*_R6Kγ_) is dependent on the *pir *gene-encoded π protein. Originally encoded by R6K, this protein is usually provided *in trans *in hosts engineered to support replication of plasmids harboring *ori*_R6Kγ_. In *Escherichia coli *this is commonly achieved by chromosomal integration of *pir *either via lysogenization with a λ*pir *phage or homologous recombination at a pre-determined locus.

**Findings:**

Current methods for construction of host strains for *ori*_R6Kγ_-containing plasmids involve procedures that do not allow selection for presence of the *pir *gene and require cumbersome and time-consuming screening steps. In this study, we established a mini-Tn*7*-based method for rapid and reliable construction of *pir*^+ ^host strains. Using a curable mini-Tn*7 *delivery plasmid, *pir *expressing derivatives of several commonly used *E. coli *cloning and mobilizer strains were isolated using both the wild-type *pir^+ ^*gene as well as the copy-up *pir-116 *allele. In addition, we isolated *pir*^+ ^and *pir-116 *expressing derivatives of a clinical isolate of *Salmonella enterica *serovar Typhimurium. In both *E. coli *and *S. enterica *serovar Typhimurium, the presence of the *pir^+ ^*wild-type or *pir-116 *alleles allowed the replication of *ori*_R6Kγ_-containing plasmids.

**Conclusions:**

A mini-Tn*7 *system was employed for rapid and reliable engineering of *E. coli *and *S. enterica *serovar Typhimurium host strains for plasmids containing *ori*_R6Kγ_. Since mini-Tn7 elements transpose in most, if not all, Gram negative bacteria, we anticipate that with relatively minor modifications this newly established method will for the first time allow engineering of other bacterial species to enable replication of plasmids with *ori*_R6Kγ_.

## Background

The γ origin of replication of the broad-host-range plasmid R6K (*ori*_R6Kγ_) has been used to construct conditionally replicative cloning and transposon delivery vectors too numerous to cite them all, with some of the most well-known vectors described in the late 1980s and early 1990s [1-6]. Replication of these vectors requires the π protein encoded by the *pir *gene which on R6K is located next to the γ origin of replication [7,8]. For maintenance of conditionally replicative plasmids that contain *ori*_R6Kγ _but lack *pir*, the π protein is expressed *in trans *from *pir *located on a compatible plasmid or, most frequently, on a λ phage or a gene inserted into the chromosome via homologous recombination at a predetermined locus [9]. In cells harboring a wild-type *pir^+ ^*gene, *ori*_R6Kγ _containing plasmids are maintained at 15 copies or less depending on size of the *ori*_R6Kγ _plasmid and *pir *gene source. A number of *pir *mutations have been identified that alter plasmid copy number, for example the *pir-116 *allele [10]. In cells harboring this allele integrated into the chromosome, *ori*_R6Kγ _plasmids are maintained at a copy number of about 250 per cell which compares to 15 copies per cell when the *pir*^+ ^allele is integrated at the same chromosomal locus [9]. The host range for *ori*_R6Kγ_-containing plasmids is limited because construction of strains supporting their replication involves methods that do not allow selection for presence of the *pir *gene and require cumbersome and time-consuming screening steps. To allow expansion of plasmid host range to customized genetic strain backgrounds we therefore developed a mini-Tn*7*-based method for rapid and reliable construction of enterobacterial *pir*^+ ^host strains.

## Results and discussion

### Development of a mini*-*Tn*7 *based *ori*_R6Kγ _chromosomal insertion system

**Figure 1:**
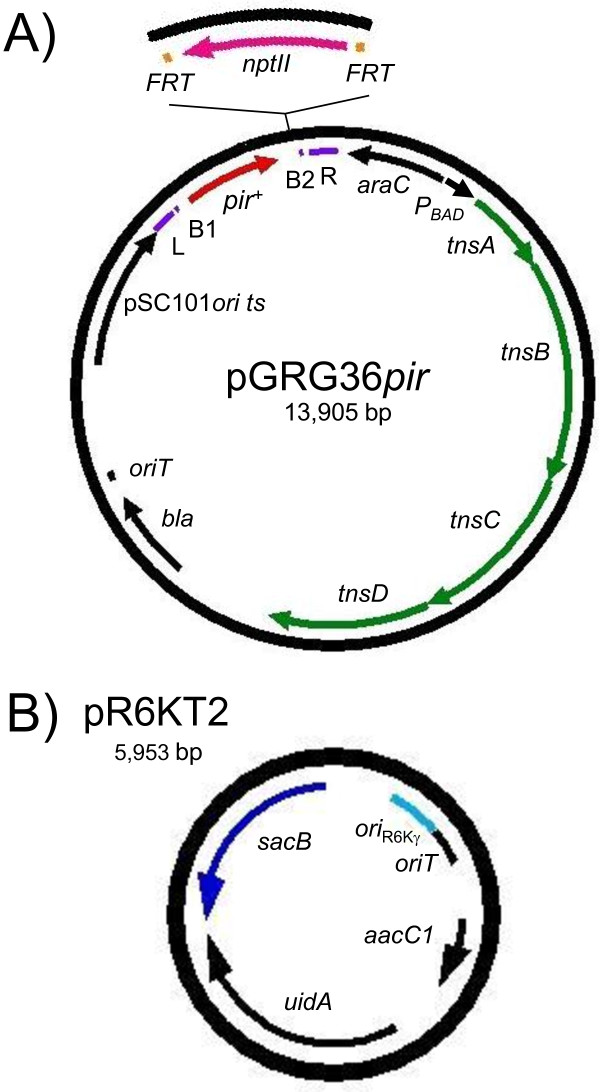
**Maps of mini-Tn*7 *delivery vector and *ori*_R6Kγ _reporter plasmid**. **A) **Map of *pir^+ ^*delivery vector pGRG36*pir*. Other versions include pGRG36*pir-116 *which contains *pir-116 *in place of *pir^+ ^*and pGRG36*pir-116-*FKm contains a *FRT*-flanked Km^r ^marker adjacent to *pir-116 *as illustrated in the inset. **B) **Map of the *ori*_R6Kγ _reporter plasmid pR6KT2. Abbreviations: *aacC1*, aminoglycoside acetyltransferase conferring gentamicin resistance; *araC*, encoding the AraC regulator of expression from the *E. coli *arabinose operon promoter *P_BAD_*; B1 and B2, *attB1 *and *attB2 *Gateway recombination sites; *bla*, β-lactamase gene conferring ampicillin resistance; *FRT*, Flp recombinase target site; L and R, Tn*7 *left and right ends; *nptII*, neomycin phosphotransferase gene conferring kanamycin resistance; *ori*_R6Kγ_, R6K plasmid γ replication origin; *oriT*, RP4 conjugal transfer origin; pSC101*ori ts*, temperature-sensitive pSC101 replication origin; *sacB*, levansucrase-encoding gene; *tnsABCD*, genes encoding the Tn*7 *site-specific transposition pathway; *uidA*, glucuronidase-encoding gene.

**Figure 2:**
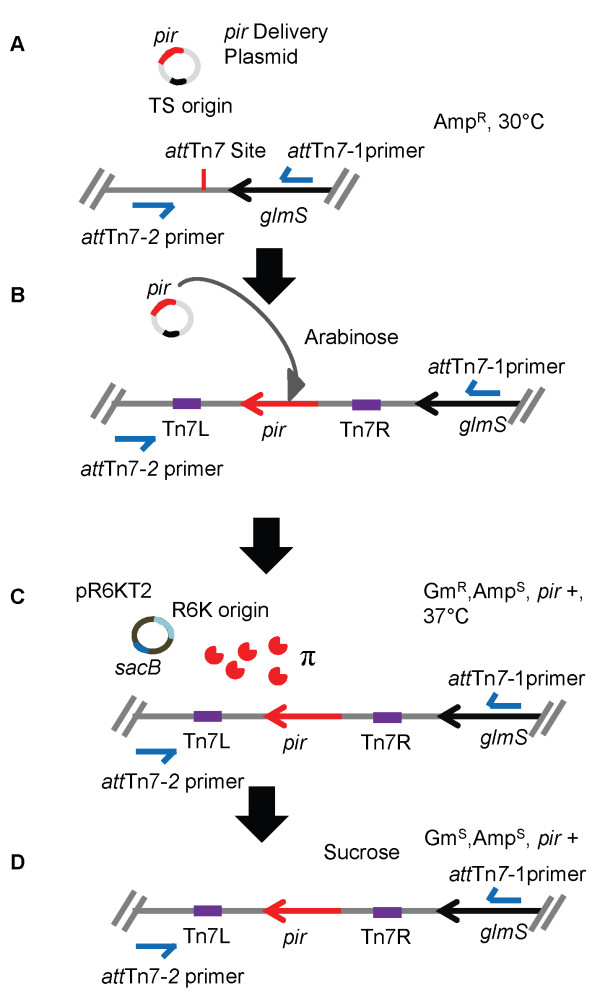
**Overview of steps involved in chromosomal mini-Tn*7*-*pir *insertion**. **A) **Introduce the mini-Tn*7*-*pir^+ ^*delivery vector by conjugation. Select ampicillin resistant (Amp^r^) colonies at 30°C to establish the delivery vector. **B) **Grow Amp^r ^cells at 30°C in presence of arabinose to induce the genes encoding the Tn*7 *site-specific transposition pathway. **C) **Introduce the *ori*_R6Kγ _reporter pR6KT2 by conjugation and grow at 37°C in the presence of gentamicin (Gm) to cure the mini-Tn*7*-*pir^+ ^*delivery vector and report integrants based on *pir*-dependent replication of pR6KT2. Establish Gm^r ^and Amp susceptible (Amp^s^) phenotype. **D) **Cure pR6KT2 reporter plasmid by plating on sucrose-containing medium. Verify Gm^s ^and Amp^s ^phenotype, and confirm *pir^+ ^*integrants by PCR (using primer pair 2372 and 2373 for *E. coli *or 2374 and 2375 for *S. enterica *serovar Typhimurium). It must be noted that PCR-based insertion site verification strategies are limited to bacteria for which sequence information about the *glmS *flanking sequences is available. Methods for identifying Tn*7 *insertion sites in bacteria for which genome sequences are unknown have been described [12].

We sought to employ the mini-Tn*7 *method described by McKenzie and Craig [11] for chromosomal insertion of *pir *alleles in the absence of selection. For this purpose, the *pir*^+ ^and *pir-116 *genes were cloned into the mini-Tn*7 *delivery vector pGRG36 (Figure 1) and published procedures [11] followed in an attempt to transpose the cloned *pir *genes into the chromosomes of various *E. coli *strains. However, in some strains, despite repetition and exhaustive PCR screening, this method proved ineffective for this purpose as the majority of colonies obtained after completion of the procedure did not contain the desired mini-Tn*7 *insertions or did not result in any insertions, for unexplained reasons. We therefore designed a method that allows positive selection of strains containing chromosomally inserted *pir *alleles (Figure 2). The rationale for this method is to establish the delivery vector with a temperature-sensitive replicon (ts), here pSC101*ori*_ts_, at permissive temperature (30°C), then introduce an *ori*_R6Kγ _reporter plasmid at 37°C, creating conditions at which the mini-Tn*7 *delivery vector is cured and replication of the reporter plasmid is dependent on the presence of a chromosomally-integrated *pir *gene. After verification of the desired mini-Tn*7*-*pir *insertions the reporter plasmid is then cured using sucrose counter-selection.

### Mini-Tn*7 *insertion of pir genes in *E. coli *and *S. enterica serovar Typhimurium*

**Figure 3:**
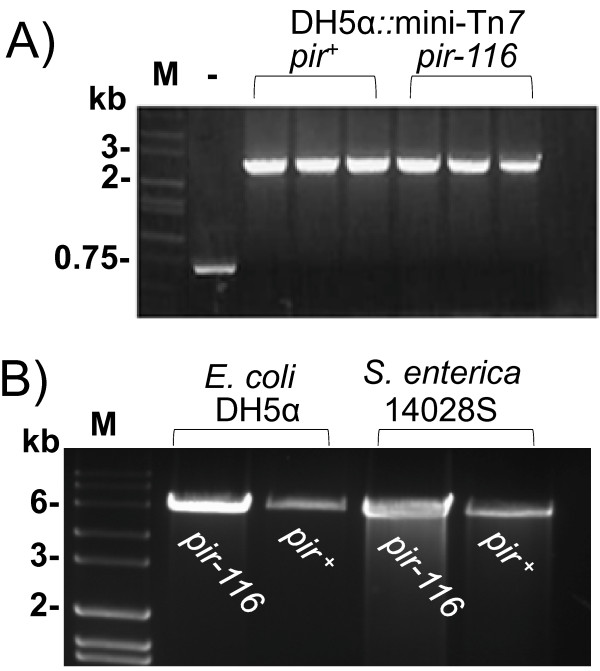
**PCR verification of mini-Tn*7*-*pir *insertion and plasmid copy number**. **A) **PCR verification of *pir^+ ^*and *pir-116 *mini-Tn*7 *insertions into *att*Tn*7 *of *E. coli *DH5α. Colony PCR with primers 2372 and 2373 (see Figure 2 for relative priming site locations) was conducted to confirm presence or absence of the mini-Tn*7*-*pir^+ ^*and mini-Tn*7*-*pir-116 *insertions. The PCR reactions were analyzed by agarose gel electrophoresis. Expected fragment sizes are 678 bp for DH5α without a mini-Tn*7 *insertion and 2,539 bp for derivatives containing mini-Tn*7*-*pir^+ ^*or mini-Tn*7*-*pir-116 *insertions. Lane M, Hi-Lo molecular size ladder from Minnesota Molecular (Minneapolis, MN) with the sizes of selected fragments indicated; Lane -, DH5α negative control (no insertion). **B) **Demonstration of pR6KT2 copy number in *pir^+ ^*and *pir-116 *containing *E. coli *and *S. enterica *serovar Typhimurium strains. Plasmid DNA was purified from overnight cultures of *E. coli *DH5α and *S. enterica *serovar Typhiumurium 14028S containing either mini-Tn7-*pir^+ ^*or mini-Tn*7*-*pir-116*. A 20 μl aliquot of each preparation was digested with *Hin*dIII to linearize the 5,953-bp plasmid and the samples were analyzed by agarose gel electrophoresis. Lane M contains the same molecular size ladder as shown in panel A and the sizes of selected fragments are indicated.

Following the procedure outlined in Figure 2, we readily obtained mini-Tn*7*-*pir *insertions in several commonly used *E. coli *laboratory cloning and mobilizer strains--DH5α, JM108, MC4100, SM10, RHO3--and a clinical *S. enterica *serovar Typhimurium isolate. Exconjugants examined by PCR contained the desired insertion, either mini-Tn*7*-*pir*^+ ^or mini-Tn*7*-*pir-116 *(Figure 3A). The plasmid copy number of pR6KT2 was greatly elevated in *pir-116*-containing *E. coli *DH5α and *S. enterica *serovar Typhimurium 14028S host strains when compared to the same strains containing chromosomally inserted wild-type *pir*^+ ^(Figure 3B). An alternative to employing an *ori*_R6Kγ _reporter plasmid is to use mini-Tn*7*-*pir *elements that contain a Km^r ^selection marker that after verification of desired inserts can be removed using *Saccharomyces cerevisiae* Flp recombinase-mediated site-specific excision, followed by curing of the Flp recombinase expression plasmid. We have successfully used this strategy in *E. coli*. Both strategies require equal time and effort.

## Conclusions

We have developed simple and effective strategies for engineering of *pir *expressing strains of Enterobacteriaceae. These strategies allow extension of the host range of *ori*_R6Kγ _containing plasmids to virtually any enterobacterial strain, something that was, to date, only possible using relatively cumbersome and time-consuming methods, e.g. isolation of λ*pir *lysogens or chromosomal insertion of cloned *pir *alleles via site-specific recombination at a pre-determined locus [9]. Mini-Tn*7 *elements insert at naturally evolved *att*Tn*7 *sites that are usually located in intergenic regions downstream of conserved *glmS *genes [12-16]. This alleviates the need for selecting potential insertion sites not affecting bacterial fitness when choosing recombinant DNA strategies for *pir *allele insertion into a bacterial genome. Since mini-Tn*7 *elements transpose in most, if not all, Gram negative bacteria, we anticipate that with relatively minor modifications this newly established method will for the first time allow engineering of other bacterial species to enable replication of plasmids with *ori*_R6Kγ_. As described, the procedure relies on availability of ts replicons which may limit its applicability to bacteria that can tolerate the non-permissive temperatures needed for plasmid curing. The methods described here were developed for *Enterobacteriaceae *which, like most bacteria, can tolerate 37°C, a temperature at which most plasmids with ts replicons, including pSC101*ori*_ts_, are readily cured. For bacteria with growth temperature optima less than 37°C, the described strategy will not work and require appropriate modifications, i.e. inclusion of different counter-selection markers, for example *sacB *[17]. Lastly, though many manipulations described in this paper use conjugations as means for introduction of plasmids into cells, some them could also be done by plasmid transformation which would alleviate the need for counter-selection strategies required for bacterial matings. We, however, consistently find that conjugations are equally convenient and more efficient means of plasmid transfer than transformation.

## Methods

### Bacterial strains, media and growth conditions

Bacterial strains used in this study are listed in Table 1. Bacteria were routinely grown at 37°C in Luria Bertani broth Lennox (LB) [18] or on LB agar purchased from MO BIO Laboratories, Carlsbad, CA. The *sacB*-containing *ori*_R6Kγ _reporter pR6KT2 was cured by plating plasmid-containing cells on yeast extract-tryptone (YT) sucrose medium containing 10 g/l yeast extract (Difco, Detroit, MI), 16 g/l tryptone (Fisher Scientific, Fairlawn, NJ), 16 g/l Bacto agar (Becton, Dickinson and Company, Sparks, MD) and 15% sucrose (w/v)[19]. Strains containing temperature-sensitive (TS) plasmid derivatives were grown at 30°C (permissive temperature) for plasmid maintenance and 37°C or 42°C (non-permissive temperature) for plasmid curing. Antibiotics were added at the following concentrations: 100 μg/ml ampicillin (Amp), 10-15 μg/ml gentamicin (Gm) and 35 μg/ml kanamycin (Km) for *E. coli *and *S. enterica *serovar Typhimurium harboring plasmids or for selection of chromosomally-integrated mini-Tn*7 *elements. Antibiotics were purchased from EMD Biosciences, San Diego, CA (Gm) and Sigma, St. Louis, MO (Amp and Km). For *E. coli *strain RHO3, media were supplemented with diaminopimelic acid (DAP; LL-, DD-, and meso-isomers; Sigma) which was added at a final concentration of 400 μg/ml for agar plates and 200 μg/ml for broth cultures. 5-bromo-4-chloro-3-indolyl-β-D-glucuronic acid (XGluc; Gold Biotechnology, St. Louis, MO) was added to media at a final concentration of 40 μg/ml. Induction of gene expression from the arabinose operon promoter (*P_BAD_*) was achieved by addition of L-arabinose to media at a final concentration of 0.5% (w/v).

**Table 1:** Bacterial strains used in this study

Bacterial strains *E. coli*	Relevant genotype	Source or reference
DB3.1	F^- ^*gyrA462 endA1 glnV44 *Δ*(sr1-recA) mcrB mrr hsdS20 *(r_B_^- ^m_B_^-^) *ara14 galK2 lacY1 proA2 rpsL20 xyl5 *Δ*leu mtl1*	Invitrogen
PIR1	F^- ^Δ(*argF-lac*)169 *rpoS*(Am) *robA1 creC510 hsdR514 endA recA1 uidA*(ΔMluI)::*pir-116*	Invitrogen
PIR2	F^- ^Δ(*argF-lac*)169 *rpoS*(Am) *robA1 creC510 hsdR514 endA recA1 uidA*(ΔMluI)::*pir^+^*	Invitrogen
RHO3	SM10 (λ*pir*)^1 ^Δ*asd*::*FRT *Δ*aphA*::*FRT*	[19]
DH5α	F^- ^ϕ80 *lacZ*ΔM15 Δ(*lacZYA-argF*)*U169 deoR recA1 endA1 hsdR17*(r_K_^- ^m_K_^+^) *phoA glnV44*	[20]
JM108	*mcrA recA1 endA1 gyrA96 thi-1 hsdR17 supE44 relA1 *Δ(*lac-proAB*)	[21]
MC4100	F^- ^*araD139 *Δ(*argF-lac*)169 *flhD5301 *Δ*(fruK-yeiR)725 (fruA25) relA1 rpsL150 rbsR22 *Δ*(fimB-fimE)632*::IS *deoC1*	[22,23]
SM10	*thi-1 thr-1 leuB26 tonA21 lacY1 supE44 recA *integrated RP4-2 Tc^r^::Mu *aphA*^+ ^(Km^r^) (RP4-2 is RP4 ΔTn*1*)	[24]
SBr1	DH5α::mini*-*Tn*7-pir^+^*	This study
SBr2	JM108::mini*-*Tn*7-pir^+^*	This study
SBr3	MC4100::mini*-*Tn*7-pir^+^*	This study
SBr4	SM10::mini*-*Tn*7-pir^+^*	This study
MaH1	DH5α::mini-Tn*7-pir-116*	This study
MaH2	JM108::mini-Tn*7-pir-116*	This study
MaH3	MC4100::mini-Tn*7-pir-116*	This study
MaH4	SM10::mini-Tn*7-pir-116*	This study
MaH5	DH5α::mini-Tn7*-pir-116-*FKm	This study
RHO4^2^	RHO3::mini-Tn*7-pir-116-*FKm	This study
RHO5^2^	RHO3::mini-Tn*7-pir-116-FRT*	This study
*S. enterica *serovar Typhimurium		
14028S	Wild Type	ATCC
SDr1	14028S::mini*-*Tn*7-pir^+^*	This study
SDr2	14028S::mini-Tn*7-pir-116*	This study

^1^The *pir *gene carried by λ*pir *has a truncation that removes the coding region for the carboxy-terminal 30 amino acids of the π protein. Despite this truncation a λ*pir *lysogen maintains plasmids with at the same copy number as cells carrying a wild-type *pir*^+ ^gene [9].

^2^The *pir-116 *allele is dominant over the *pir *gene carried by the lysogenic λ phage and leads to increased copy number of *ori*_R6Kγ _containing plasmids.

## DNA and genetic methods

### Isolation of plasmid DNA

Plasmid DNAs were isolated from *E. coli *and *S. enterica *serovar Typhimurium by using a Fermentas GeneJET Plasmid MiniPrep Kit (Fermentas, Glen Burnie, MD).

### Transposition of mini-Tn*7*

The respective mini-Tn7 delivery vectors were transformed into *E. coli *mobilizer strain RHO3 [19]. Conjugation of delivery plasmid into *E. coli *and *S. enterica *serovar Typhimurium strains was achieved by biparental mating using previously described methods [19] with some minor modifications. Briefly, RHO3 donor and *E. coli *and *S. enterica *serovar Typhimurium recipient cultures were grown overnight at 30°C (pGRG36-based donor strains) or 37°C (recipient strains) in LB medium with the appropriate nutritional (DAP) and antibiotic (Amp) supplements for RHO3 with the mini-Tn*7 *delivery vector. One ml of donor and recipient were placed into separate 1.7 ml microcentrifuge tubes and harvested by centrifugation in a microcentrifuge for 30 s at 13,400×*g *and room temperature. Cells were washed twice in 1 ml LB medium and then re-suspended in 200 μl of LB medium. Equal volumes (25 μl) of each cell suspension were transferred to a cellulose acetate membrane (13 mm diameter; 0.45 μM pore size; Sartorius Stedim, Bohemia, NY) sitting on an LB agar plate containing 400 μg/ml DAP and 0.5% arabinose. After overnight incubation at 30°C, the membrane was transferred to a microcentrifuge tube containing 1 ml of LB and cells dislodged by centrifugation in a microfuge for 30 s at 13,400×*g *and room temperature. After removing the membrane, cells were washed twice in 1 mL LB and then re-suspended in 200 μl of LB medium. The entire sample was placed on an LB-agar plate with 100 μg/ml Amp and 0.5% arabinose, and a portion streaked for single colonies with an inoculating loop. The plates were incubated at 30°C overnight or until single colonies were clearly discernable.

A single purified colony was then used as recipient for the *ori*_R6Kγ _reporter pR6KT2. This plasmid was introduced via biparental mating from RHO3 as described above, except that recipient cells were grown in the presence of Amp and arabinose and RHO3/pR6KT2 cultures were grown in the presence of Gm and DAP. After overnight incubation at 30°C mating mixtures were recovered and plated on LB with 15 μg/ml Gm at 37°C to cure the temperature-sensitive mini-Tn*7 *delivery vector and select for pR6KT2. Purified colonies were patched on LB, LB + Gm and LB + Amp to confirm the loss of the mini-Tn*7 *delivery vector (Amp susceptibility) and presence of pR6KT2 (Gm resistance). After verification of mini-Tn*7 *insertions by PCR, pR6KT2 was cured by streaking a single colony on YT medium with sucrose and XGluc, and incubating overnight at 37°C. Single colonies were patched on LB and LB + Gm plates to confirm the loss of the plasmid. The *pir *gene insertions in the resulting strains were then re-confirmed by PCR and sequencing of the resulting DNA fragments.

When using the mini-Tn*7*-*pir*-FKm vectors, the protocol for conjugation was as described above for mini-Tn*7 *delivery without antibiotic selection. Exconjugants were grown overnight in LB + DAP and arabinose at 30°C, and Km^r ^transposon insertions were selected by plating conjugation mixtures on LB plates with 35 μg/ml Km followed by incubation at 42°C to cure the delivery plasmid. The Km^r ^marker can optionally be deleted from the strain with the mini-Tn*7*-*pir*-FKm insertion by transformation with pFLP2 (or any other Flp recombinase-expressing plasmid such as pCP20 [25]), testing transformants for Km susceptibility and then curing pFLP2 by plating on sucrose-containing media following previously described protocols [17].

### Confirmation of mini-Tn*7-pir *insertions

Insertions of mini-Tn*7*-*pir *at *att*Tn*7 *in *E. coli *and *S. enterica *serovar Typhimurium were performed by colony PCR using DNA in boiling preparation lysates as templates. These lysates were obtained by transferring separate colonies to individual sterile microcentrifuge tubes containing 30 μl of sterile water and boiling for 10 min. Using 6 μl of these boiling preparations and *Taq *DNA polymerase from New England Biolabs, PCR reactions were performed in a total volume of 50 μl. Primer pairs 2372 (5'-GATGCTGGTGGCGAAGCTGTC) & 2373 (5'-GATGACGGTTTGTCACATGGAG) and 2374 (5'-CAGCAACGACAACATGCACA) & 2375 (5'-AAACCATCAGCGCGGAACAA) were used for *E. coli *and *S. enterica *serovar Typhimurium, respectively. In each case, the entire PCR reaction was analyzed on a 1% agarose gel. Expected PCR fragment sizes are 678 bp for *E. coli *without a mini-Tn*7 *insertion and 2,539 bp for derivatives containing mini-Tn*7*-*pir*^+ ^and mini-Tn*7*-*pir-116*. Fragment sizes for *S. enterica *serovar Typhimurium without and with mini-Tn*7*-*pir *insertions are 485 bp and 2,345 bp, respectively. When utilizing mini-Tn*7*-*pir*-FKm, the fragment sizes obtained with strains containing *pir*^+ ^or *pir-116 *insertions change by +1,470 bp when the Km^r ^marker is present or by +145 bp after its Flp-mediated excision.

### Plasmid construction

Plasmids used in this study are listed in Table 2. The Gateway-compatible mini-Tn*7 *delivery vector pGRG36GW was constructed by cloning of a 1,770-bp *Stu*I-*Xho*I fragment from pUC18-mini-Tn7T-Gm-GW [16] between the *Sma*I and *Xho*I sites of pGRG36 [11], followed by transformation into the *gyrA462 *strain DB3.1.

**Table 2:** Plasmids used in this study

Plasmids	Description	Reference/Source
pGRG36^a^	Amp^r^; mini-Tn*7 *delivery vector with TS replicon	[11]
pGRG36GW	Amp^r^, Cm^r^; Gateway version of pGRG36	This study
pDONR221	Cm^r^, Km^r^; Gateway cloning vector	Invitrogen
pDONR221*pir*	Km^r^; *pir^+ ^*donor vector	This study
pDONR221*pir-116*	Km^r^; *pir-116 *donor vector	This study
pGRG36*pir*	Amp^r^; *pir^+ ^*delivery vector	This study
pGRG36*pir-116*	Amp^r^; *pir-116 *delivery vector	This study
pGRG36*pir-116*-FKm	Amp^r^, Km^r^; *pir-116*-FKm delivery vector	This study
pFKM4	Amp^r^, Km^r^; pFKM2 [26] with *Xba*I and *Spe*I sites removed and *Pac*I site introduced	This study
pR6KT2	Gm^r^; Suc^s^; *ori*_R6Kγ _reporter plasmid	This study

^a^The DNA sequence of pGRG36 can be obtained from GenBank under accession number DQ460223. Sequences of other plasmids constructed in this study are available from the authors upon request.

The *pir*^+ ^and *pir-116 *Gateway donor clones were constructed as follows. First, the *pir^+ ^*and *pir-116 *genes were PCR-amplified from *E. coli *strain PIR2 (*pir*^+^) and PIR1 (*pir-116*) chromosomal DNA templates using HiFi *Taq *polymerase (Invitrogen) and primers 1333 (5'-TGAGCGTCGCAGAACATTACA) & 1334 (5'-ACCTGGGTGGACGATATCAC). The resulting 1,264-bp PCR fragments were cloned into the TA cloning vector pCR2.1 (Invitrogen) to create pCR2.1*pir *and pCR2.1*pir-116*. Next, *attB *sequences were attached to the respective *pir *gene segments using PCR with primers 1558 (5'-GGGGACAAGTTTGTACAAAAAAGCAGGCTTGAGCGTCGCAGAACATTACA; the *attB1 *sequence is underlined) & 1559 (5'-GGGGACCACTTTGTACAAGAAAGCTGGGTACCTGGGTGGACGATATCAC; the *attB2 *sequence is underlined), and pCR2.1-*pir *and pCR2.1-*pir-116 *DNA as templates. This generated 1,323-bp DNA fragments that were recombined into pDONR221 using Gateway BP clonase reactions which created pDONR221*pir *and pDONR221*pir-116*, respectively. Finally, the inserts from pDONR221*pir *and pDONR221*pir-116 *were transferred to pGRG36GW using LR Gateway recombination to yield pGRG36*pir *and pGRG36*pir-116*. The pGRG36*pir-116*-FKm plasmid was constructed by cloning a 1,470-bp *Sal*I fragment from pFKM4 containing a Km^r ^gene flanked by Flp recombinase target (*FRT*) sites into the *Xho*I site of pGRG36*pir-116*.

The *ori*_R6Kγ _reporter pR6KT2 was constructed as follows. First, the *ori*_R6Kγ _and *oriT *regions were PCR-amplified from pUC18R6KT-mini-Tn*7*T [16] using *Taq *polymerase (NEB) and primers 2298 (5'-ATTCCCGGGAGGCCACCACTTCAAGAACTC) & 2299 (5'-TAATCCCGGGCTTCCGCTTCCTCGCTCA). The resulting 824-bp amplicon was cloned into

pCR2.1 to create pCR2.1-R6KoriT. Second, the 901-bp *Xba*I fragment from non-methylated pPS856 [17] DNA obtained by isolating the plasmid from *dam dcm E. coli *JM110 [21] was ligated with the 6.5-kb *Spe*I and *Xba*I digested backbone of pEXKm5 [19] to construct pEXGm5B. Third, the 915-bp *ori*_R6Kγ _and *oriT Xba*I and *Eco*53K1 fragment was released from pCR2.1::R6K*oriT *and ligated with the 6.2-kb *Xba*I and *Sma*I digested pEXGm5B backbone to create pR6KTSCE. Finally, pR6KTSCE was digested with *Bmg*B1 and *Fsp*I and the 5.9-kb backbone was re-circularized to construct the reporter vector pR6KT2.

All plasmid constructions were verified by restriction digest and DNA sequence analysis.

## Competing interests

The authors declare that they have no competing interests.

## Authors' contributions

BK and SB performed most of the experiments, JP, IM, EB, SL, KM and KHC contributed to plasmid construction and performed pilot experiments, BK and RKS designed and supervised experiments, and BK, RKS and HPS drafted the final manuscript. All authors read and approved the final manuscript.
